# A structural connectivity atlas of limbic brainstem nuclei

**DOI:** 10.3389/fnimg.2022.1009399

**Published:** 2023-01-12

**Authors:** Simon Levinson, Michelle Miller, Ahmed Iftekhar, Monica Justo, Daniel Arriola, Wenxin Wei, Saman Hazany, Josue M. Avecillas-Chasin, Taylor P. Kuhn, Andreas Horn, Ausaf A. Bari

**Affiliations:** ^1^Department of Neurosurgery, David Geffen School of Medicine at the University of California, Los Angeles, Los Angeles, CA, United States; ^2^Stanford Department of Neurosurgery, Stanford University, Palo Alto CA, United States; ^3^Department of Radiology, VA Greater Los Angeles Healthcare System, David Geffen School of Medicine at UCLA, Los Angeles, CA, United States; ^4^Department of Neurosurgery, University of Nebraska Medical Center, Omaha, NE, United States; ^5^Department of Psychiatry and Biobehavioral Sciences, University of California, Los Angeles, Los Angeles, CA, United States; ^6^Movement Disorder and Neuromodulation Unit, Department of Neurology, Charité–Universitätsmedizin Berlin, Corporate Member of Freie Universität Berlin and Humboldt–Universität zu Berlin, Berlin, Germany; ^7^Department of Neurology, Center for Brain Circuit Therapeutics, Harvard Medical School, Brigham and Women's Hospital, Boston, MA, United States; ^8^Massachusetts General Hospital Neurosurgery and Center for Neurotechnology and Neurorecovery (CNTR) at MGH Neurology Massachusetts General Hospital, Harvard Medical School, Boston, MA, United States

**Keywords:** brainstem, deep brain stimulation, limbic system, tractography, atlas

## Abstract

**Background:**

Understanding the structural connectivity of key brainstem nuclei with limbic cortical regions is essential to the development of therapeutic neuromodulation for depression, chronic pain, addiction, anxiety and movement disorders. Several brainstem nuclei have been identified as the primary central nervous system (CNS) source of important monoaminergic ascending fibers including the noradrenergic locus coeruleus, serotonergic dorsal raphe nucleus, and dopaminergic ventral tegmental area. However, due to practical challenges to their study, there is limited data regarding their *in vivo* anatomic connectivity in humans.

**Objective:**

To evaluate the structural connectivity of the following brainstem nuclei with limbic cortical areas: locus coeruleus, ventral tegmental area, periaqueductal grey, dorsal raphe nucleus, and nucleus tractus solitarius. Additionally, to develop a group average atlas of these limbic brainstem structures to facilitate future analyses.

**Methods:**

Each nucleus was manually masked from 197 Human Connectome Project (HCP) structural MRI images using FSL software. Probabilistic tractography was performed using FSL's FMRIB Diffusion Toolbox. Connectivity with limbic cortical regions was calculated and compared between brainstem nuclei. Results were aggregated to produce a freely available MNI structural atlas of limbic brainstem structures.

**Results:**

A general trend was observed for a high probability of connectivity to the amygdala, hippocampus and DLPFC with relatively lower connectivity to the orbitofrontal cortex, NAc, hippocampus and insula. The locus coeruleus and nucleus tractus solitarius demonstrated significantly greater connectivity to the DLPFC than amygdala while the periaqueductal grey, dorsal raphe nucleus, and ventral tegmental area did not demonstrate a significant difference between these two structures.

**Conclusion:**

Monoaminergic and other modulatory nuclei in the brainstem project widely to cortical limbic regions. We describe the structural connectivity across the several key brainstem nuclei theorized to influence emotion, reward, and cognitive functions. An increased understanding of the anatomic basis of the brainstem's role in emotion and other reward-related processing will support targeted neuromodulatary therapies aimed at alleviating the symptoms of neuropsychiatric disorders.

## Highlights

– The brainstem plays a key role in the processing of emotional stimuli and is intricately linked with the limbic system.
– Anatomic data for these connections is limited in humans.
– We describe the structural connectivity of five brainstem nuclei (locus coeruleus, ventral tegmental area, periaqueductal grey, dorsal raphe nucleus, and nucleus tractus solitarius) in relation to limbic circuits.
– Our results present a comprehensive delineation of the brainstem-limbic structural connectivity of these nuclei and are compiled into a freely available tractographic atlas.
– Applications include future targeting of these structures for neuropsychiatric conditions.

## Introduction

The brainstem, comprised of the medulla oblongata, pons, and midbrain, comprises ~3% of the mass of the brain and contains about 2% of the neurons in the central nervous system. Yet, what it lacks in size it makes up for in complexity and a disproportional influence on processes ranging from autonomic functions to arousal and consciousness (Azevedo et al., 2009; Herculano-Houzel, 2009). The brainstem provides vital autonomic regulation and homeostatic maintenance. It also serves as a conduit for all fibers linking the cerebral cortex and cerebellum to the spinal cord. Furthermore, it functions as a major afferent sensory system, receiving input from visceral fibers and cranial nerve nuclei which it then filters and transmits (often through several intermediary nuclei), to higher cortical centers.

The brainstem also plays a key role in emotional processing. Three brainstem networks have been identified that are thought to contribute to limbic processing: (1) the ascending sensory network consisting of the spinothalamic tracts, medial forebrain bundle, nucleus of the tractus solitarius (NTS), parabrachial nuclear complex and thalamic nuclei, (2) the descending motor network consisting of the periaqueductal grey (PAG), caudal raphe nucleus, and locus coeruleus (LC), and (3) the modulatory network with the serotonergic dorsal raphe (DRN), noradrenergic LC, and dopaminergic ventral tegmental area (VTA) (Angeles Fernández-Gil et al., 2010; Venkatraman et al., 2017). The ascending, descending and modulatory brainstem networks allow for the progressive integration and processing of information as signals move rostrally through the brainstem, thalamus and then to the cortex, but also carry information in reverse, with cortical regions regulating the action-response relationships of phylogenetically older structures (Tucker et al., 2002).

**Figure 1:**
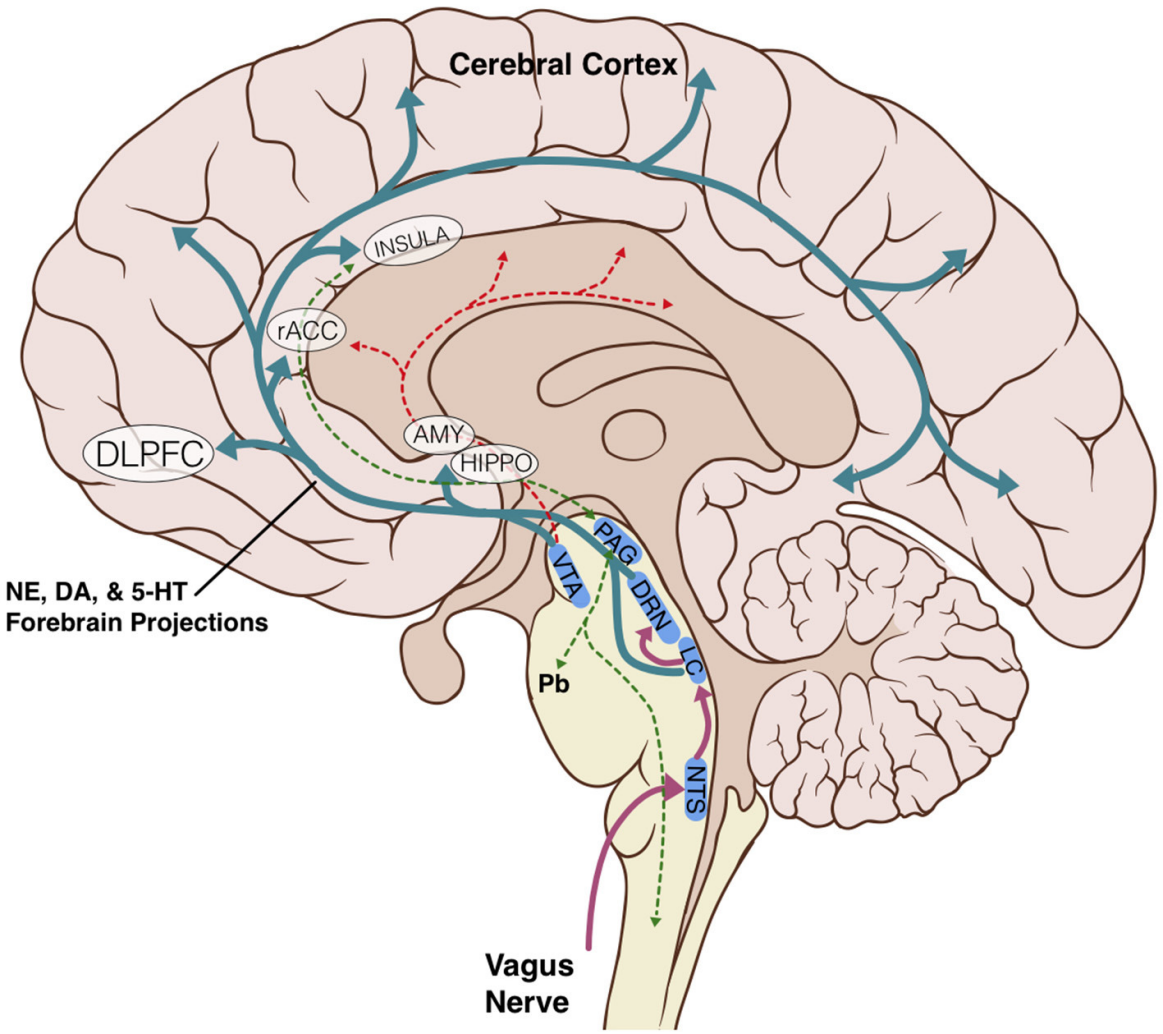
Schematic representation of key brainstem-cortex circuits. DLPFC, dorsolateral-prefrontal cortex; rACC, rostral anterior cingulate cortex; INSULA, insular cortex; AMY, amygdala; HIPPO, hippocampus; VTA, ventral tegmental area; PAG, periaqueductal grey; DRN, dorsal raphe nucleus; LC, locus coeruleus; NTS, nucleus tractus solitarius; Pb, parabrachial nucleus; NE, norepinephrine; DA, dopamine; 5-HT, serotonin.

The anatomic and structural basis for the brainstem's role in limbic processing is theorized to involve several key nuclei which are the sole or major source of potent monoamine neurotransmitters for the higher cerebral cortex: the LC (norepinephrine), DRN (serotonin), and VTA (dopamine). Additionally, the PAG and NTS serve as inputs or centers of modulation to these monoamine neural networks (Figure 1). Several monoamine neurotransmitters have, individually or in combination, been implicated in disease states including Parkinson's disease, major depressive disorder, or addiction, and are essential for physiological activities including arousal, sleep/wake cycles, perception of pain, affect, and goal directed behavior. Existing evidence obtained largely from animal studies (Table 1) describes how these brainstem nodes interact with cortical limbic structures to convey body state and homeostatic information to result in behaviors such as heightened alertness, arousal from sleep, fear, and defense measures (Omar et al., 2008; Ulrich-Lai and Herman, 2009). Lesion studies have also demonstrated the emotional role of the brainstem utilizing models or subjects with disease states such as Alzheimer's Disease, brainstem-cerebellar pathology, and brainstem infarcts (van Zandvoort et al., 2003; Omar et al., 2008; Mariën and D'aes, 2015; Dutt et al., 2021). However, despite their importance, it has been challenging to study brainstem nuclei in humans *in vivo* because of difficulties in accurately defining the nuclei on imaging (Edlow et al., 2012; Song et al., 2014; Tang et al., 2018). Therefore, limited anatomic data exists for these brainstem-limbic relationship in humans.

**Table 1:** Limbic brainstem nuclei key characteristics.

**Nuclei**	**Cell Type**	**Key points**
Nucleus tractus solitarius (NTS) Owen et al. (2008), Weinstein et al. (2015), and George et al. (2019)	Multiple	The Nucleus Solitarius is the major afferent nuclei for vagal visceral sensory fibers and the subsequent relay of that information to other brainstem centers (notably the LC and DRN) and eventually higher cortical centers. It is directly involved in the vagal nerve stimulation pathway.
Locus coeruleus (LC) Levy et al. (1987), Sillery et al. (2005), and Adell, 2015; Sims-Williams et al. (2017)	Norepinephrine	The Locus Coeruleus-Norepinephrine (LC-NE) system plays a key role in arousal, attention, and stress responses. The LC is the sole source of NE to cortical circuits. The LC-NE system is greatly impacted in neurodegenerative diseases such as Parkinson's Disease, which is theorized to result from abnormal signaling leading to cognitive and motor manifestations of the disease. It also received direct input from the NTS and has been shown to be essential for the efficacy of VNS in epilepsy.
Dorsal raphe nucleus (DRN) Arias-Carrión and Pöppel (2007) and Settell et al. (2017)	Serotonin	Major source of serotonin that project to the forebrain. Receives projections from the LC and, some argue, from the NTS as well. Lesions in rats abolish the efficacy of VNS for epilepsy.
Periaqueductal Grey (PAG) Fischl et al. (2002); Angeles Fernández-Gil et al. (2010), and Venkatraman et al. (2017)	Multiple	The PAG is a key structure in pain modulation, sympathetic responses, and the learning of defensive and aversive behaviors. As a result, the PAG is important in everyday interactions with the environment and human responses to adversive stimuli. The PAG is thought to contribute to defensive behaviors, panic attacks, anxiety, depression, and migraines.
Ventral tegmental area (VTA) Angeles Fernández-Gil et al. (2010), Ogisu et al. (2013), and Venkatraman et al. (2017)	Dopamine	Processing of reward and aversive experiences, major source of dopamine for mesocortical-limbic pathway.

There are two general approaches to defining regions of interest (ROI) on MRI, manual and automated segmentation. Automated segmentation has been demonstrated to reliably delineate cortical structures (Fischl et al., 2002; Woolrich et al., 2009). It has also been used successfully to define certain regions of the brainstem (particularly for studies interested in masking the medulla, pons and midbrain separately) (Patenaude et al., 2011; Iglesias et al., 2015; Wang et al., 2016; Sander et al., 2019), yet it remains technically difficult to define most individual brainstem nuclei *via* this method. While there are several automated techniques, most recent attempts have been to mask brainstem nuclei using convolutional neural network-based segmentation, a deep learning image recognition technique, to delineate the substantia nigra (Berre et al., 2019) and LC (Dünnwald et al., 2020). However, this method relies on neuromelanin-MRI scans, limiting its application to non-pigmented regions. Furthermore, other techniques such as voxel intensity-based algorithms still require some manual delineation and thresholding and can be complicated by homogenously intense regions (Ogisu et al., 2013; Zhang et al., 2016; Berre et al., 2019; Dünnwald et al., 2020). Therefore, although automated processing technologies are being developed, manual segmentation still has significant advantages and is considered the gold standard.

Manual segmentation is labor intensive, often resulting in studies with small sample sizes and high inter-rater variability. In addition, utilizing a manually-defined mask as an atlas for new subjects can be more computationally intensive than automated segmentation techniques (Aljabar et al., 2009; Berre et al., 2019). Yet, a well-trained individual, given proper anatomic knowledge and tools, can produce reliable results and manually delineated atlases are still considered by many to be the gold standard (Aljabar et al., 2009; Morey et al., 2009; Iglesias et al., 2015).

In this study, we manually segmented five brainstem nuclei to perform probabilistic tractography to selected limbic targets in 197 human subjects. We hypothesized that autonomic and monoamine brainstem nuclei would demonstrate structural connectivity to cortical limbic regions, namely the amygdala, insula and hippocampus. Additionally, since there were no existing atlases of the structural connectivity of these nuclei, we aimed to produce an accurate anatomical atlas for use in future research.

We overcame some of the challenges of performing tractography on the brainstem by using a rigorous anatomic definition scheme with a combination of voxel measurements and anatomic landmarks to reduce variability while defining each nucleus. Additionally, we were able to achieve a scale of nearly 200 subjects, similar to many automated based approaches, thereby increasing the power of the study and reducing the effects of outliers or isolated errors. Lastly, we obtained diffusion MRI (dMRI) scans from the Human Connectome Project which provided a large dataset with scans acquired in a high resolution allowing for increased accuracy (Glasser et al., 2013; Van Essen et al., 2013). Overall, our results present a comprehensive delineation of the brainstem-limbic structural connectivity of these nuclei and are compiled into a freely available tractographic atlas.

## Methods

### Subjects

Data were obtained from the publicly available WU-Minn HCP 1,200 Subjects data release repository (Glasser et al., 2013; Van Essen et al., 2013). The scanning protocol was approved by Human Research Protection Office (HRPO), Washington University (IRB# 201 204 036). No human subject experimental procedures were undertaken at the authors' home institutions. The participants included in the HCP 1,200 Subjects data release provided written informed consent as approved by the Washington University IRB. From this repository, 200 total non-twin subjects were randomly selected. The analysis was limited to these subjects based on available computational resources. Three subjects were excluded due to lack of required imaging data files. The remaining 197 subjects were included in our analyses and a description of their demographic characteristics is provided in Table 2.

**Table 2:** Cohort.

**Gender**	***n***	**Average age (range)**	**Handedness** **(-100 = L, +100 = R)**	**Years of education**
Female	101	29.6 (22–35)	76.5	15.1
Male	96	27.4 (22–36)	60.3	14.9
Total	197	28.5 (22–36)	68.6	15.0

Gender, average age education and handedness of participant. Note that handedness was measured using the Edinburgh Handedness questionnaire (Oldfield, 1971). Negative numbers indicate that a subject is more left-handed than right-handed, while positive numbers indicate that a subject is more right-handed than left-handed.

### MRI acquisition

The data were acquired on a modified Siemens 3T Skyra scanner with a customized protocol^21^. The T1-weighted MRI has an isotropic spatial resolution of 0.7 mm, and the dMRI data have an isotropic spatial resolution of 1.25 mm. The multi-shell dMRI data were collected over 270 gradient directions distributed over three b-values (1,000, 2,000, 3,000 s/mm^2^). For each subject, the multi-shell dMRI data were collected with both L/R and R/L phase encodings using the same gradient table, which were then merged into a single copy of multi-shell dMRI data after the correction of distortions with the HCP Preprocessing Pipeline. Average T1w and T2w images were then aligned to MNI space (with 0.7 mm resolution), with 6 degrees of freedom which also aligns with AC-PC line and the inter-hemispheric plane but which does not alter the original size and shape of the brain. Acquisition time was 32 min for T1w scans which the majority of the atlas used here was based on [FOV = 224 mm, matrix = 320, 256 sagittal slices in a single slab, TR=24,00 ms, TE = 2.14 ms, TI = 1,000 ms, FA=8°, Bandwidth (BW) = 210 Hz per pixel] (Glasser et al., 2013; Sotiropoulos et al., 2013).

### Masking of seed structures and anatomic boundaries

The areas of interest in this study consisted of brainstem structures with widespread projections. Due to the wide inter-subject anatomic variation and the small nature of many of these structures, all seed masks were created manually for each subject on the original Human Connectome Project (HCP) structural MRI scans using FSLeyes software. The seed masks generated in this fashion included the Locus Coeruleus (LC), Nucleus Tractus Solitarius (NTS), Periaqueductal Grey (PAG), Dorsal Raphe Nucleus (DRN) and Ventral Tegmental Area (VTA).

**Figure 2:**
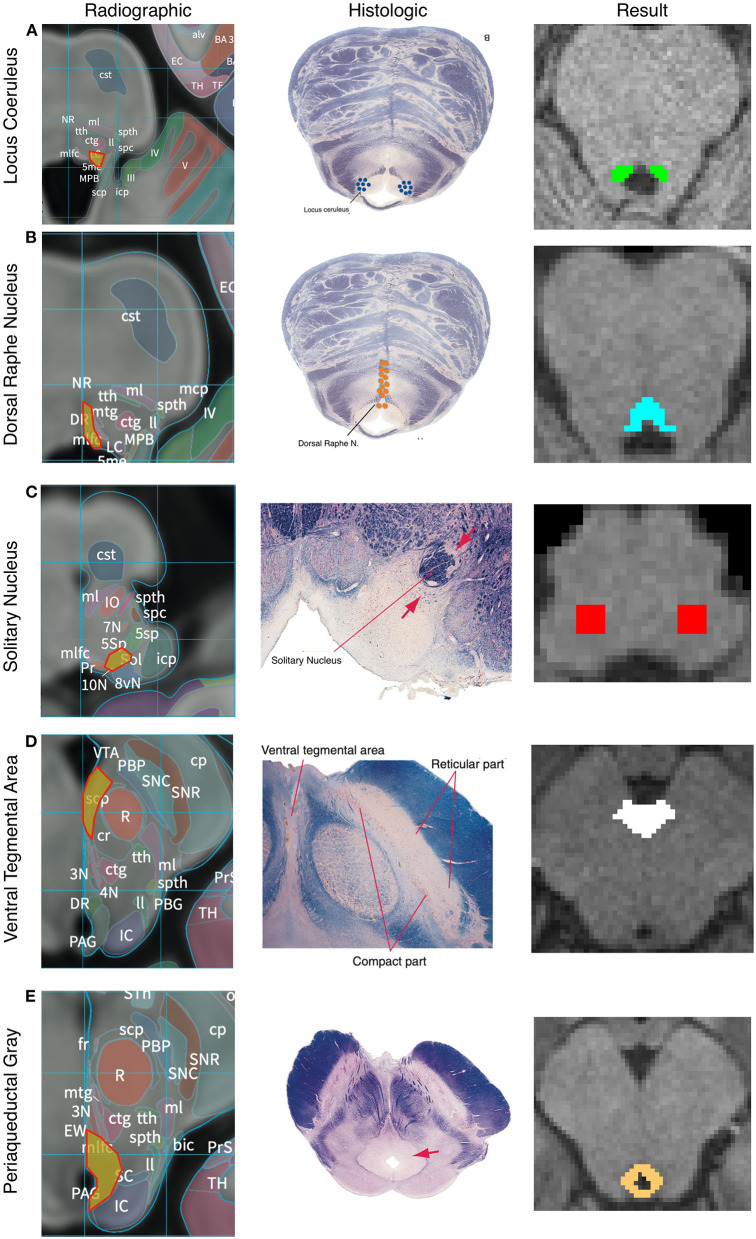
Anatomic boundaries. Diagram of how anatomic boundaries of nuclei were masked. First radiographic^23^ **(left pane)** and histologic atlas^24^ **(middle pane)** were carefully examined for each nucleus. Using a combination of anatomic landmarks and voxel measurements (not shown), the resulting mask **(right pane)** was created in FSL. Locus Coreleus **(A)**, Dorsal Raphe Nucleus **(B)**, Solitary nucleus **(C)**, Ventral tenemental area **(D)**, Periaquaductal Grey **(E)**.

The anatomic boundaries used for each set of masks are described below and shown in Figure 2 and Anatomic Supplement 1. Multiple histologic and radiographic resources were used to carefully define each region of interest (Desikan et al., 2006; Naidich et al., 2009; Edlow et al., 2012; Mai et al., 2016; Vanderah, 2019). Individual raters were first trained on a sample data set and their accuracy was assessed relative to a template mask. Raters had to achieve an error rate of <5% to begin masking subjects. To ensure a high degree of internal consistency, no more than two individuals were responsible for creating the masks of each nucleus. We utilized a two expert review method where an expert neuroradiologist and neurosurgeon evaluated each mask based on the below standardized anatomic boundaries. This provided internal validity. After review between the two expert raters, there was agreement that all masks were within their tolerance as described by the anatomic protocols in Figure 2. Between 30 and 60 min were spent per subjects creating, editing, and reviewing the 5 seed masks.

After initial masking was completed, the fslmaths *boxv* command was used to dilate each mask by a factor of 5 followed by an erosion of 5 to ensure edges were smoothed and gaps were filled.

### Locus coeruleus

The LC was defined in both the caudal-rostral and medial-lateral planes. The most caudal point was the midpoint of the 4^th^ ventricle and the most rostral was the rostral pons. Laterally, the LC was defined to be 3 mm lateral to the midline at the anterior-lateral angle of the 4^th^ ventricle (Figure 2A). Average volume of the seed mask was 94.5 mm^3^.

### Dorsal raphe nucleus

Beginning at the caudal midbrain, the DRN was masked caudally until its termination in the mid pons. The cerebral aqueduct and fourth ventricle were used as guides with the rostral portions measuring 12 voxels wide tapering to 6 voxels at the most caudal aspect (Figure 2B). Average volume of the seed mask was 153.8 mm^3^.

### Nucleus tractus solitarius

The caudal aspect of the NTS was defined to begin 3 mm rostral to the obex. It then proceeded rostrally in a “V” shape. Standardized voxel measurements were used to ensure a consistent shape across individual. The NTS was masked caudally until the middle cerebellar peduncles were clearly visible on a horizontal section. Since the NTS also tracks slightly more anteriorly as it progresses rostrally, the anterior-caudal boundary was defined in the horizontal plane as the point where a line would transect from the root of cranial nerve VIII, with the point halfway between the midpoint and lateral aspect of the 4^th^ ventricle on either side of the pons (Figure 2C). Average volume of the seed mask was 207.3 mm^3^.

### Ventral tegmental area

The most inferior transverse section of the VTA was defined at the section of the midbrain where the interpeduncular fossa opens to the interpeduncular cistern. The VTA boundary on inferior sections was the medial border of the Substantia Nigra. The lateral and medial borders were directly adjacent to the interpeduncular fossa. As the VTA progresses rostrally it becomes a contiguous structure with its medial boundaries joining in the midline bordering the medial aspect of the interpeduncular fossa and extending in a posterior direction to the midpoint of the medial edge of the red nucleus. The most superior section of the VTA was defined at the level of the mammillary bodies. Standardized voxel measurements were used to ensure a consistent shape across individual subjects (Figure 2D). Average volume of the seed mask was 214.1 mm^3^.

### Periaqueductal grey

From a mid-coronal slice, the aqueduct was identified on the sagittal perspective. On the axial perspective, the lateral ventricles were traced inferiorly until it became the cerebral aqueduct which delineated the superior margin of the mask. A diamond-shaped border surrounding the aqueduct was demarcated as the PAG on the axial plane. The inferior border of the mask was determined to be the location where the mammillary bodies became fully defined, which correlated to the opening of the fourth ventricle. Standardized voxel measurements were used to ensure a consistent shape across individual subjects (Figure 2E). Average volume of the seed mask was 172.2 mm^3^.

### Probabilistic tractography

Probabilistic tractography was carried out using FSL's FMRIB Diffusion Toolbox (probtrackx) with modified Euler streaming (Woolrich et al., 2009; Jenkinson et al., 2012). We used the diffusion tensor model that was fitted on processed diffusion data. ROI were delineated using standard FSL parameters (specified at http://fsl.fmrib.ox.ac.uk/fsl/fslwiki/) to derive fractional anisotropy maps for each ROI. The bedpost command was used to generate an estimate of crossing fiber orientation at the level of individual voxels. Then, utilizing a so called “ball and stick” model, we selected 3 sticks and left all other options as default. Importantly, at each voxel, all possible fiber orientations were considered taking into account standard assumptions about uncertainty when computing whether a given set of voxels will be considered to be “in-line” so as to count as a streamline. For more detail on the underlying algorithms please see these references (Behrens et al., 2003, 2007; Woolrich et al., 2009).

Target masks were generated using the Harvard-Oxford subcortical atlas (Desikan et al., 2006). Target masks included the amygdala (AMY), dorsolateral prefrontal cortex (DLPFC), hippocampus, insula, Nucleus accumbens (NAc), orbitofrontal cortex (OFC), and rostral anterior cingulate cortex (rACC). Seed masks also served as target masks once produced such that the number of targets increased overtime as new seeds were created. All tractography was performed between each (right and left) seed and the ipsilateral target. Each target mask was also a termination mask such that tractography was terminated once a streamline entered the target. Additionally, Freesurfer standard lookup tables were used to generate Ipsilateral white matter masks which were used as waypoints. The ventricles and cerebellum masks were similarly generated with Freesufer and used as exclusion masks. We used the “onewaycondition,” curvature 0.2, 2,000 samples, steplength = 0.5, fibthresh = 0.01, distthresh = 1 and sampvox = 0.0. This resulted in 14 or more seed_to_target output files representing a voxelwise map of the number of seed samples from each seed to target.

To calculate the probability of connectivity (POC) between each brainstem seed voxel to each of the 7 cortical and to the other 4 brainstem nuclei targets, we ran the FSL proj_thresh subroutine with a threshold of 1,250 on each probtrackx output. For each voxel in the seed mask with a value above the threshold, *proj_thresh* calculates the number of samples reaching each of the target masks as a proportion of the total number of samples reaching any of the target masks. The fsl waytotal and waynorm commands were used to normalize the tractography results by seed and target size. This yielded a separate map of each seed mask for each target with each voxel having a value between 0 and 1 representing the POC of that voxel to the given target. Thus, there were 2 maps (one for each hemisphere) per seed and per target for each subject. To produce an overall POC from each seed mask to target, probabilities were averaged across all voxels in each map. We next created a population connectivity map across all 197 subjects. Each of the previously created proj_thresh maps was registered to MNI 1 mm standard space, thresholded at a level of 0.1 and binarized. These maps were then added across all 197 subjects such that each voxel value now represented the number of subjects with connectivity to the target.

### Parallel data processing

FSL software was implemented in a distributed fashion using Amazon Web Services (AWS, http://aws.amazon.com) EC2 instances running in parallel. Each AWS EC2 instance was an r4.large clone of an Amazon Machine Image (AMI) running Ubuntu 14.04 with FSL software version 5.0.10. FSL bedpostx directories for each subject and the probtrackx output files were stored on an Amazon S3 bucket.

### Statistical analysis

Statistical analysis was carried out using the R software package (http://www.r-project.org/) and Prism 8 software from Graphpad (https://www.graphpad.com/).

To analyze the structural data, the subject specific output from the proj_thresh files stored on Amazon S3 were downloaded to a new EC2 instance running Ubuntu 14.04.1. FSLmaths was then used to compute the mean connectivity to each target using fslstats. The means were then imported to RStudio (version 1.3.959) running R (version 3.6.3) and the means to each target region were compared with a single factor analysis of variance (ANOVA). A Tukey HSD test was then run to determine statistical significance of the variance in the means.

Overall connectivity measurements were obtained by first taking the average of right and left connectivity for each subject specific seed-target relationship and then computing the mean across all subjects.

### MNI atlas

Separately, each seed mask was warped to MNI space using the FSL *applywarp* command and the HCP subject specific nonlinear acpc_dc2standard file. The MNI152 NLIN 2009b T1 0.5 mm brain was used as reference (ICBM 152 Nonlinear atlases, 2009). Once warped, each mask was averaged across all 197 subjects and set the threshold to two standard deviations from the mean to exclude extreme values.

## Results

Summative images in MNI space are publicly available at: https://www.uclahealth.org/neurosurgery/research-areas. Additionally, the atlas comes preinstalled on the widely used neurostimulation software, *Lead-DBS* (https://www.lead-dbs.org/helpsupport/knowledge-base/atlasesresources/atlases). All other data is available upon request of the corresponding author.

**Figure 3:**
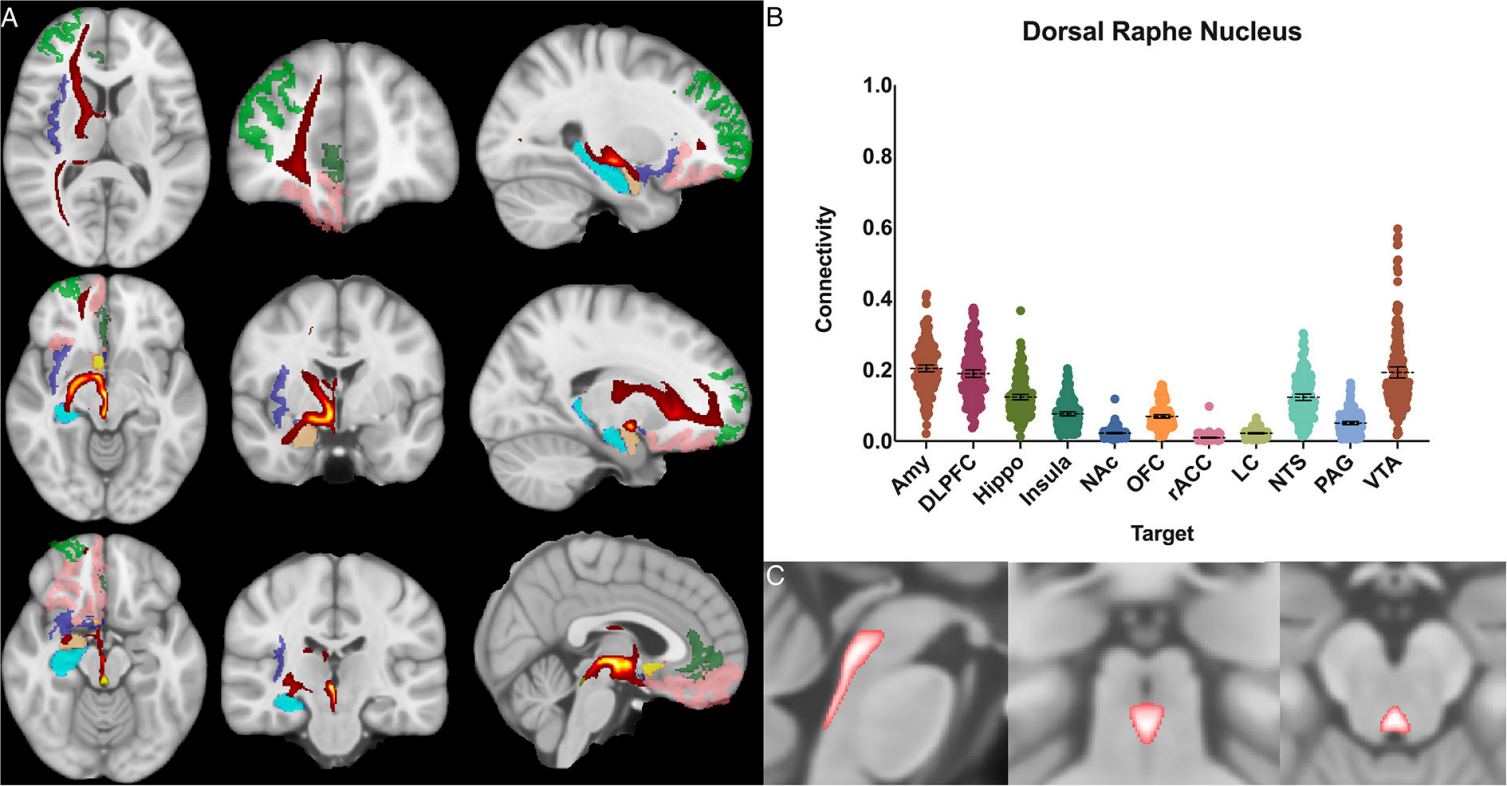
Dorsal raphe nucleus structural results. **(A)** MNI space structural connectivity results visual representation averaged over all 197 subjects. Brighter yellow on heat map indicates a high number of samples passing through a given point that will eventually reach a target map (brighter yellow = more samples). Dark green: DLPFC; pink: OFC; brown: AMY; blue: HIPPO; purple: insula; orange: NAc; light green: rACC. **(B)** Mean connectivity results with dashed line showing mean and 95% CI, each point on graph shows result from individual subject. **(C)** Anatomic MNI mask of seed region.

**Figure 4:**
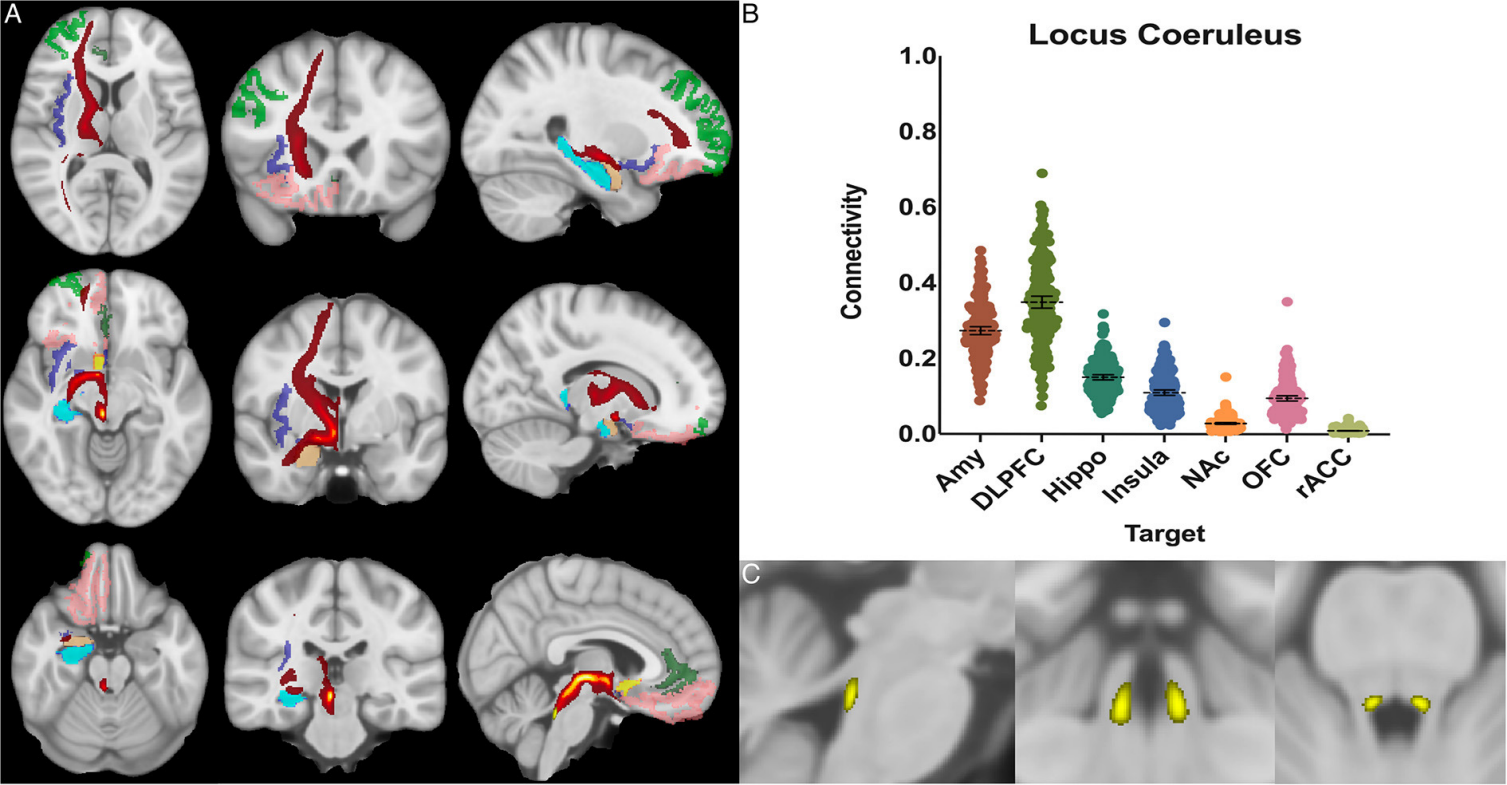
Locus coeruleus structural results. **(A)** MNI space structural connectivity results visual representation averaged over all 197 subjects. Brighter yellow on heat map indicates a high number of samples passing through a given point that will eventually reach a target map (brighter yellow = more samples). Dark green: DLPFC; pink: OFC; brown: AMY; blue: HIPPO; purple: insula; orange: NAc; light green: rACC. **(B)** Mean connectivity results with dashed line showing mean and 95% CI, each point on graph shows result from individual subject. **(C)** Anatomic MNI mask of seed region.

**Figure 5:**
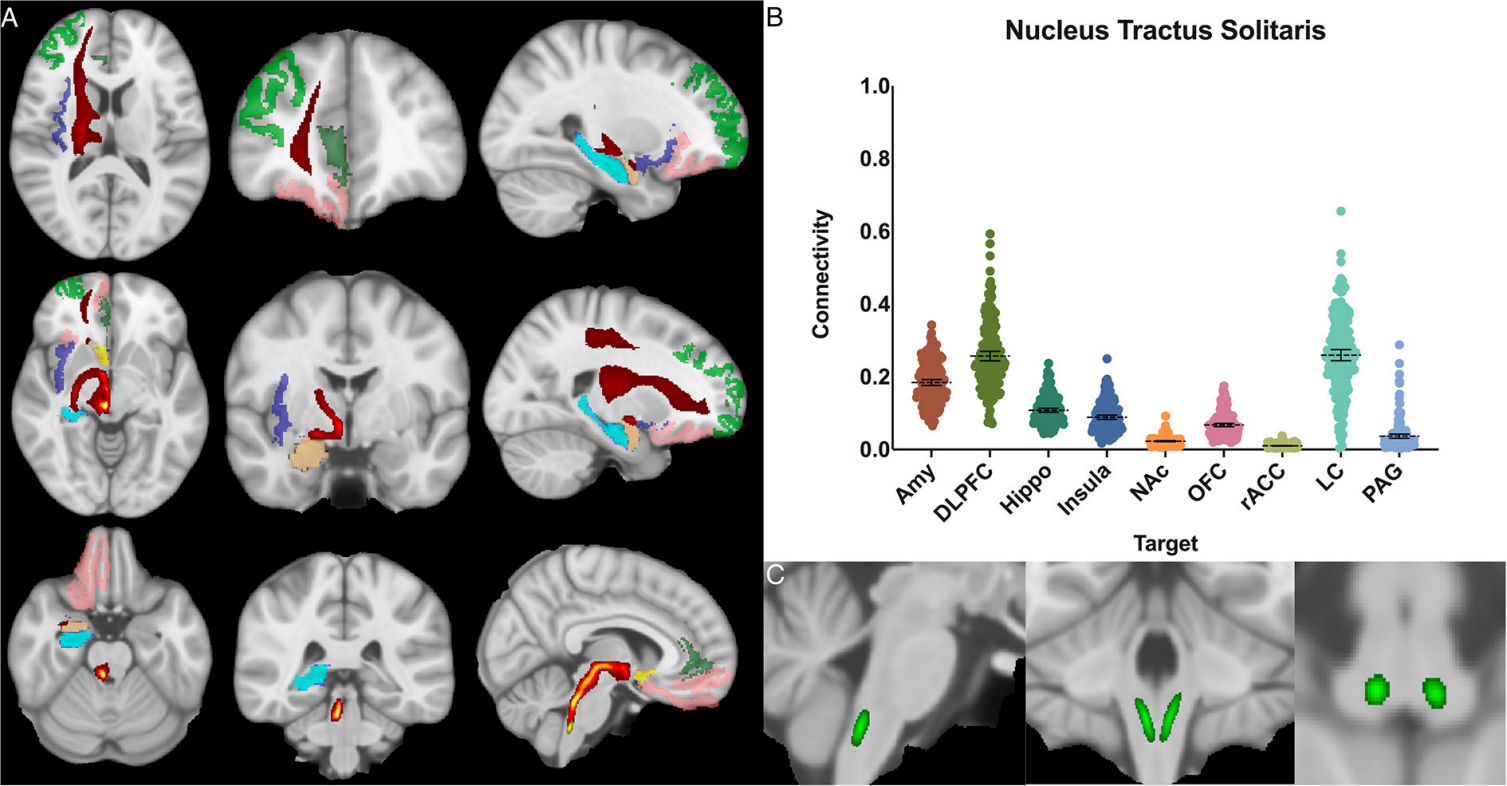
Nucleus tractus solitarius structural results. **(A)** MNI space structural connectivity results visual representation averaged over all 197 subjects. Brighter yellow on heat map indicates a high number of samples passing through a given point that will eventually reach a target map (brighter yellow = more samples). Dark green: DLPFC; pink: OFC; brown: AMY; blue: HIPPO; purple: insula; orange: NAc; light green: rACC. **(B)** Mean connectivity results with dashed line showing mean and 95% CI, each point on graph shows result from individual subject. **(C)** Anatomic MNI mask of seed region.

**Figure 6:**
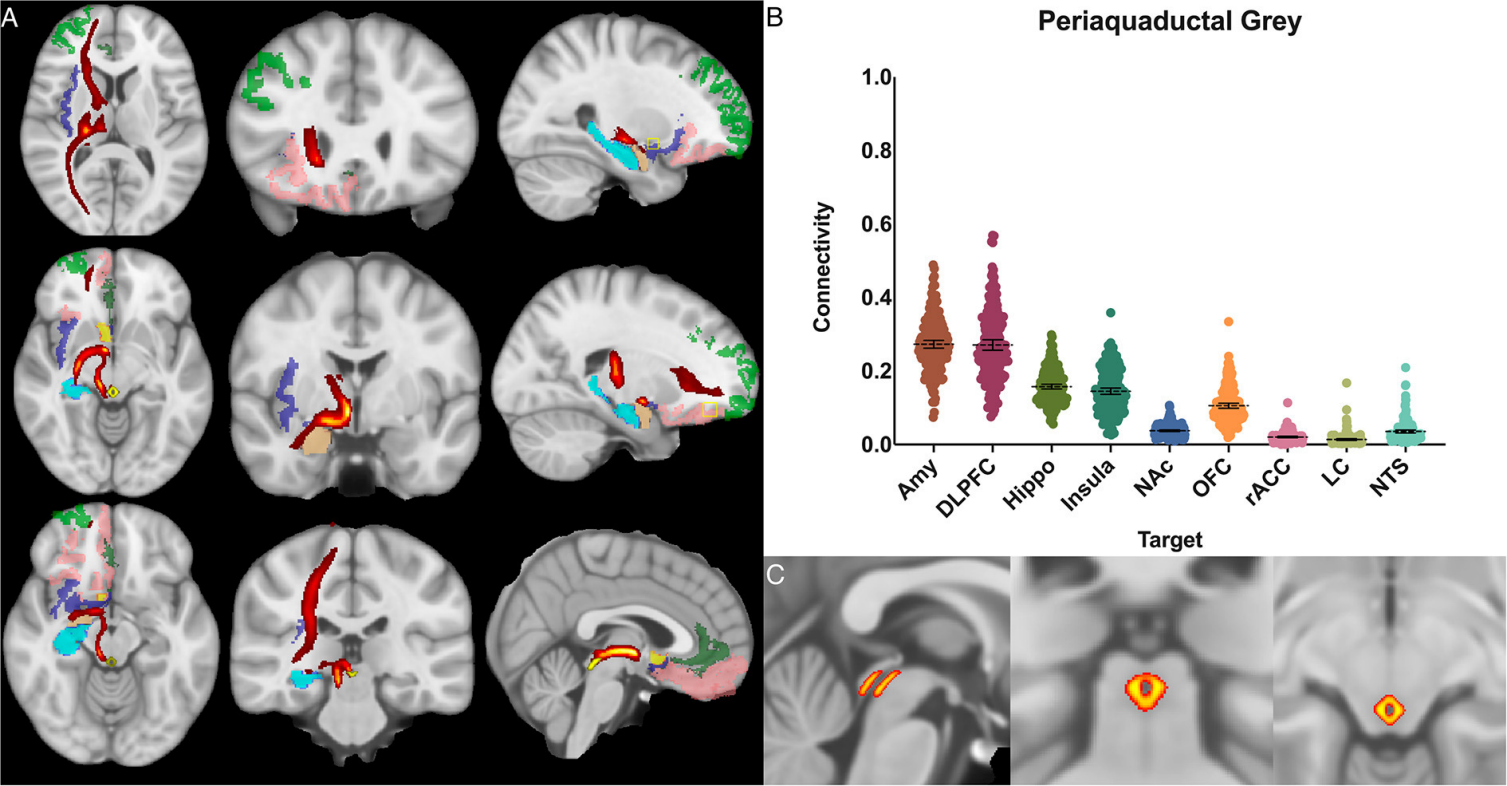
Periaqueductal grey structural connectivity. **(A)** MNI space structural connectivity results visual representation averaged over all 197 subjects. Brighter yellow on heat map indicates a high number of samples passing through a given point that will eventually reach a target map (brighter yellow = more samples). Dark green: DLPFC; pink: OFC; brown: AMY; blue: HIPPO; purple: insula; orange: NAc; light green: rACC. **(B)** Mean connectivity results with dashed line showing mean and 95% CI, each point on graph shows result from individual subject. **(C)** Anatomic MNI mask of seed region.

**Figure 7:**
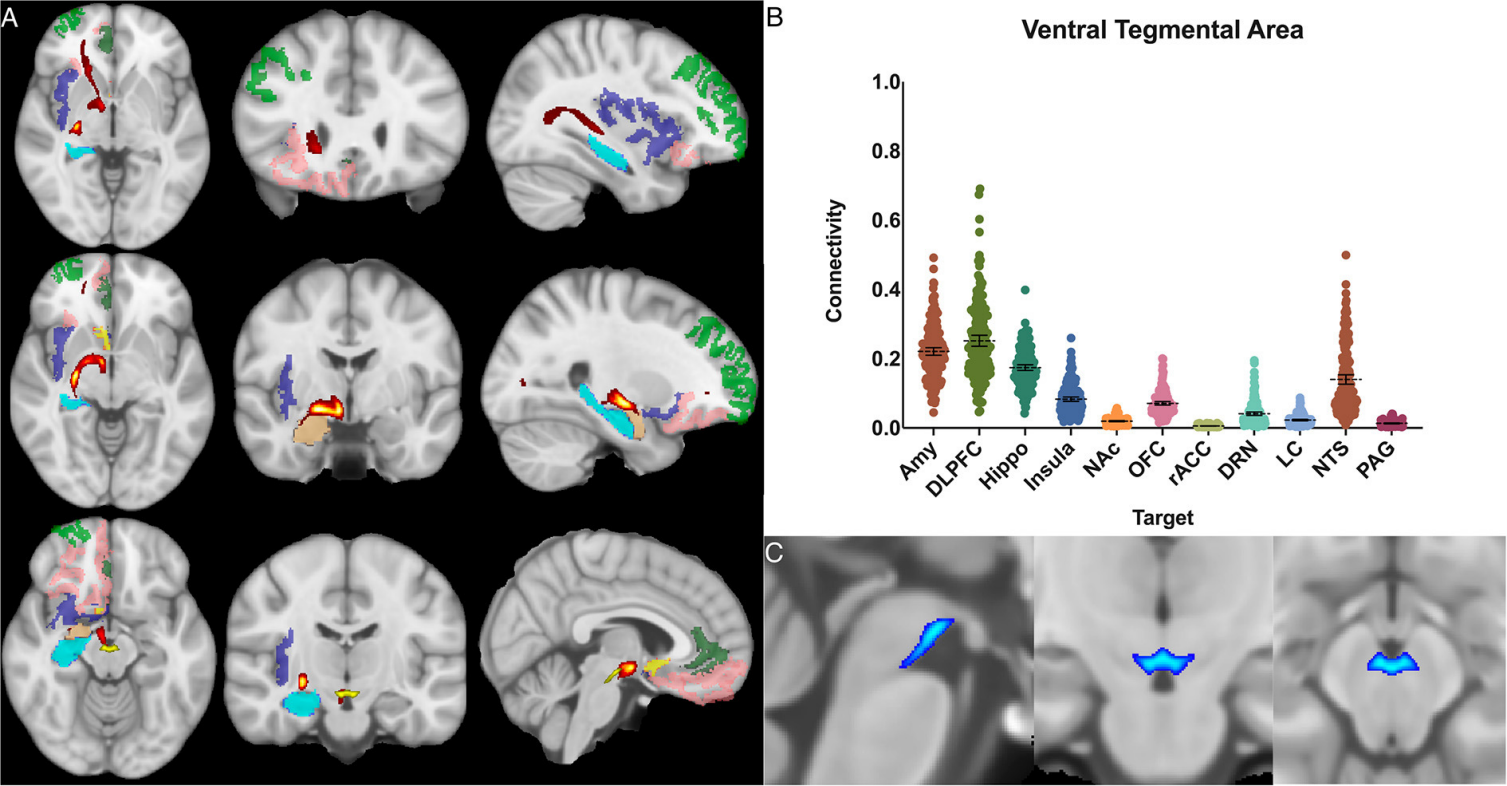
Ventral tegmental area structural connectivity. **(A)** MNI space structural connectivity results visual representation averaged over all 197 subjects. Brighter yellow on heat map indicates a high number of samples passing through a given point that will eventually reach a target map (brighter yellow = more samples). Dark green: DLPFC; pink: OFC; brown: AMY; blue: HIPPO; purple: insula; orange: NAc; light green: rACC; **(B)** Mean connectivity results with dashed line showing mean and 95% CI, each point on graph shows result from individual subject. **(C)** Anatomic MNI mask of seed region.

**Figure 8:**
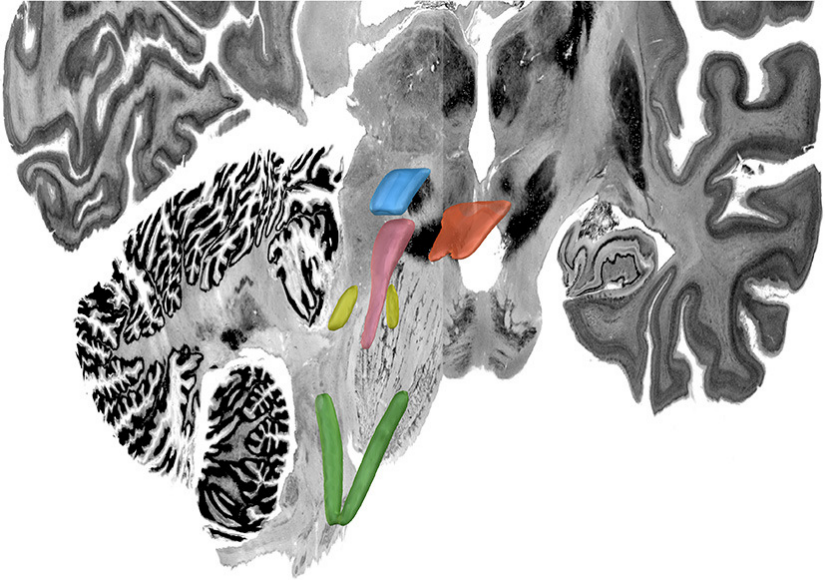
MNI 3D Atlas. Green: nucleus tractus solitaris; yellow: locus coeruleus; pink: periaqueductal grey; blue: ventral tegmental area; orange: dorsal raphe nucleus. For this figured, Lead-DBS was used to visualize the same volumetric MNI seed masks as shown in Figures 3–7 which are here overlaid on the MNI template brain to provide a 3-D representation of the relationship between these brainstem nuclei.

Complete results for the average POC for each seed to target relationship is reported in Table 3. Figures 3–7 details the average streamline paths, provides a graphic representation of mean connectivity, and portrays the anatomic boundaries of each seed mask averaged over all subjects in MNI space. The three highest POCs are reported here for each seed. A summative three-dimensional atlas generated with *Lead-DBS* utilizing an MNI-152 0.5 mm template brain is also showed (Figure 8).

**Table 3:** Average probabilities of connectivity; format: Mean [95% CI interval].

	**Dorsal raphe nucleus**	**Locus coeruleus**	**Nucleus tractus solitarius**	**Periaqueductal grey**	**Ventra tegmental area**
Amygdala	0.204 [0.195, 0.214]	0.273 [0.262, 0.284]	0.184 [0.176, 0.192]	0.273 [0.262, 0.283]	0.221 [0.210, 0.232]
Dorsolateral prefrontal cortex	0.189 [0.179, 0.200]	0.349 [0.333, 0.364]	0.257 [0.244, 0.270]	0.271 [0.257, 0.285]	0.252 [0.236, 0.268]
Hippocampus	0.124 [0.116, 0.131]	0.150 [0.143, 0.157]	0.108 [0.102, 0.113]	0.157 [0.151, 0.164]	0.174 [0.166, 0.182]
Insula	0.077 [0.071, 0.082]	0.110 [0.102, 0.117]	0.089 [0.083, 0.095]	0.145 [0.136, 0.154]	0.083 [0.077, 0.089]
Nucleus accumbens	0.023 [0.021, 0.024]	0.028 [0.026, 0.031]	0.023 [0.021, 0.024]	0.038 [0.035, 0.040]	0.020 [0.018, 0.021]
Orbital prefrontal cortex	0.070 [0.065, 0.074]	0.095 [0.088, 0.102]	0.067 [0.063, 0.027]	0.106 [0.099, 0.112]	0.071 [0.066, 0.076]
Rostra anterior cingulate cortex	0.010 [0.008, 0.011]	0.009 [0.009, 0.010]	0.010 [0.009, 0.011]	0.020 [0.018, 0.022]	0.006 [0.005, 0.006]
Dorsal raphe nucleus	-	-	-	-	0.041 [0.036, 0.046]
Locus coeruleus	0.022 [0.020, 0.024]	-	0.260 [0.244, 0.275]	0.014 [0.011, 0.016]	0.023 [0.021, 0.025]
Nucleus tractus solitarius	0.123 [0.114, 0.132]	-	-	0.035 [0.031, 0.039]	0.140 [0.126, 0.154]
Periaqueductal grey	0.051 [0.046, 0.055]	-	0.037 [0.031, 0.042]	-	0.013 [0.012, 0.015]
Ventral tegmental area	0.193 [0.177, 0.208]	-	-	-	-

Within each seed region, ANOVA with multiple comparisons (ANOVA-MC) and *post-hoc t-*test decompositions demonstrated significant (adjusted p<0.05) difference in the POC between each seed and target structure except as noted below. The only other exceptions to this were if the total POC was <0.03 for both structures.

The dorsal raphe nucleus demonstrated average POC to each target as follows: AMY 0.204 [95% CI 0.195, 0.214], VTA 0.193 [95% CI 0.177, 0.208], DLPFC 0.189 [95% CI 0.179, 0.200]. However, ANOVA-MC did not demonstrate a significant difference between these structures (Figure 3).

The locus coeruleus demonstrated the greatest POC to the DLPFC 0.349 [95% CI 0.333, 0.364], followed by the AMY 0.273 [95% CI 0.262, 0.284] and Hippocampus 0.150 [95% CI 0.143, 0.157] (Figure 4).

The nucleus tractus solitarius showed the greatest POC to the LC 0.260 [95% CI 0.244, 0.275], followed by the DLPFC 0.257 [95% CI 0.244, 0.270] and the AMY 0.184 [95% CI 0.176, 0.192]. There was no significant difference between NTS-LC and NTS-DLPFC POC (Figure 5).

For the periaqueductal grey the highest POC was to the AMY 0.273 [95% CI 0.262, 0.283], followed by DLPFC 0.271 [95% CI 0.257, 0.285], however this difference was not significant (*P* > 0.99). The third greatest POC was to the hippocampus at 0.157, significantly less than either the AMY (*p* < 0.001) or the DLPFC (*p* < 0.001) [95% CI 0.151, 0.164] (Figure 6).

The ventral tegmental area showed the greatest POC to the DLPFC 0.252 [95% CI 0.236, 0.268], AMY 0.221 [95% CI 0.210, 0.232], and the hippocampus 0.174 [95% CI 0.166, 0.182]. Of note, there was no significant difference between VTA-DLPFC and VTA-AMY POC (*p* = 0.26) (Figure 7).

**Figure 9:**
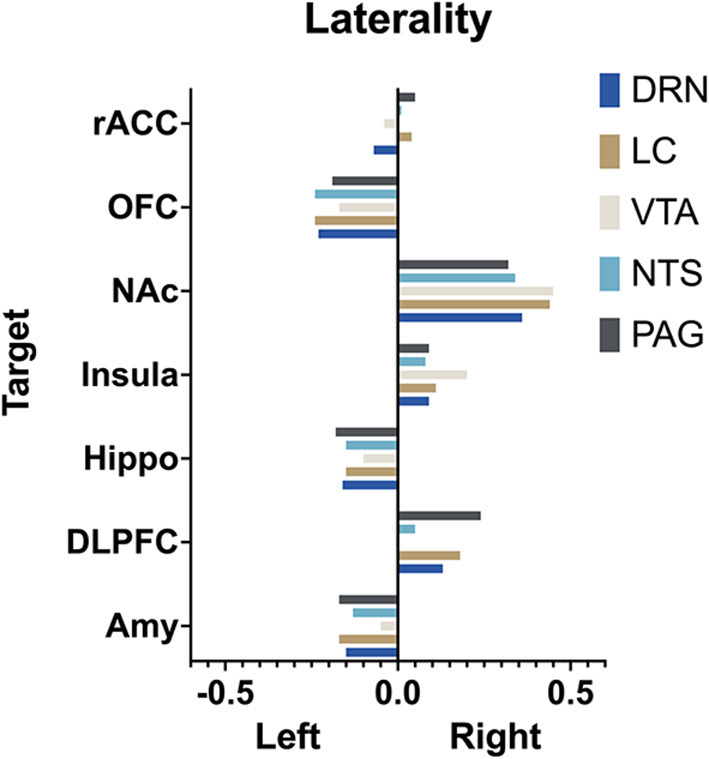
Laterality. Arbitrary measure of laterality by seed nuclei. **(Left)** is set as negative and right as positive. More extreme values indicate greater difference in mean connectivity between **(left)** and **(right)**.

The extent to which each nucleus demonstrated laterality of connectivity was also calculated. The mean connectivity for each seed to target relationship was computed for the left and right and the difference between left and right was divided by the total connectivity to give a relative value for laterality. Left was given a negative value and right a positive value. Interestingly, there was no significant difference (*p* > 0.05, two way ANOVA with multiple comparison) between the mean laterality for each seed nuclei regardless of which target was selected (Figure 9).

The demographic characteristics of the data set was homogenous by design (Table 2). The *t-*test was performed between each seed-target POC and compared between males (*n* = 96) and females (*n* = 101). *P* > 0.05 for all relations except for the PAG-insula connection with male POC = 0.135 and female POC = 0.154 (*P* = 0.025).

## Discussion

In this study, we carried out manual *in vivo* segmentation of defined brainstem regions of interest in a large cohort of subjects and performed probabilistic tractography between these regions and several limbic target regions of interest in the study of emotion and reward. The seeds selected were the NTS, LC, DRN, VTA, and PAG. These were chosen because of their clinical significance, the importance and wide-ranging effects of their respective neurotransmitters, and their established role in emotion and reward processing. We evaluated the connectivity between each of these seeds individually to a set of limbic processing centers to test the hypothesis of whether there would be a high POC between brainstem nuclei and these regions. Lastly, we aggregated these results into an MNI atlas and have made this publicly available to all interested researchers. This data represents a unique comprehensive *in vivo* analysis of key brainstem nuclei in a large human population and is also one of the first such studies to examine the relationship of these structures utilizing the Human Connectome Project.

Overall, our connectivity analysis of these five brainstem nuclei supports the role of the brainstem in emotional processing and confirmed our hypothesis that these brainstem nuclei demonstrate structural connectivity with known limbic regions. There is strong evidence that most mammals have analogous experiences to human emotion, even with far less evolved higher cortical centers, indicating that important processing occurs at levels below the cortex (Craig, 2003; Omar et al., 2008; Angeles Fernández-Gil et al., 2010; Holstege, 2016; Venkatraman et al., 2017). In order to further understand the basis of emotion and reward, and intervene in cases of disorders of emotion and reward processing, it is critical to understand phylogenetically older structures such as the brainstem and recognize them as critical factors in emotional processing.

Emotions can be thought of as mental representations associated with distinct sensory states with the evolutionary goal of producing relevant behavioral responses in an organism (Venkatraman et al., 2017). It is therefore logical that the brainstem is a key modulator of this system as it is able to receive afferent visceral and somatic sensory information, begin filtering and processing these signals, and transmit them to higher cortical centers. Existing literature suggests that many of these processes are related to the monoamine neuropeptides, norepinephrine, serotonin, and dopamine. These neurotransmitters are produced by these brainstem nuclei which then project to higher cortical centers (Holstege, 1992; Plutchik, 2001; Edlow et al., 2012; Venkatraman et al., 2017). Networks and nodes in the brainstem are increasingly considered key aspects of the conscious experience of emotion and therefore are of great clinical significance to neuropsychiatric disorders (Edlow et al., 2012, 2015; Venkatraman et al., 2017).

In our analysis, each seed region demonstrated its own pattern of connectivity largely consistent with existing anatomical information from other imaging, postmortem and animal studies (Naidich et al., 2009; Edlow et al., 2015, 2019; Bianciardi et al., 2016; Mai et al., 2016; Venkatraman et al., 2017; Tang et al., 2018). A general trend was observed for high probabilities of connectivity with the amygdala, DLPFC, and hippocampus, with relatively lower connectivity with the OFC, NAc, insula, and rACC, suggesting that while there is heterogeneity among these nuclei, there is also a distinct pattern of brainstem connectivity with both frontal and temporal lobe structures.

### Dorsal raphe nucleus

The neurons which transmit serotonin and project to the cerebral cortex are mainly clustered around the dorsal and rostral aspects of the raphe nucleus (Hornung and De Tribolet, 1995). The results here pertain only to the rostral portion as the largely inferiorly projecting caudal half was selectively excluded in the anatomic boundaries for this analysis (Figure 2B). The serotonin system is widely studied and is known to modulate fear and anxiety and other social behaviors (Zangrossi et al., 2001; Moskowitz et al., 2003; Arbib and Fellous, 2004; Gobrogge et al., 2017). Treatments for depression and anxiety uses medications selective serotonin reuptake inhibitors (SSRIs) targeted at this system (Adell, 2015; Pillai et al., 2019). In animals, serotonergic projection to the amygdala and hippocampus has been associated with anxiety and retrieval of fear memories, and this relationship is under study in humans as a possible mechanistic explanation for major depressive disorder (Lowry et al., 2008; Dayan and Huys, 2009; Ohmura et al., 2010; Weinstein et al., 2015; Brakowski et al., 2017; Anand et al., 2018).

Based on histological data, the DRN's projection patterns are complex and include temporal lobe structures such as the hippocampus and amygdala, the PAG, LC, and frontal and insular cortices as well (Hornung, 2003). Functional connectivity studies have shown alterations in DRN connectivity to the frontal and cingulate cortices in subjects with depression (Weinstein et al., 2015; Anand et al., 2018). Given the DRN's significance in depression, there have been studies analyzing DRN-amygdala structural connectivity (Schmaal et al., 2016; Pillai et al., 2019). While, the DRN has been included in whole-brain brainstem connectivity studies which demonstrated a connectivity profile congruent with the histological evidence described above (Bianciardi et al., 2016), to our knowledge, this is the first study to focus specifically on the structural connectivity of the DRN to the limbic system in humans.

We found significant cortical connectivity of the DRN to the amygdala (0.20), DLPFC (0.19), hippocampus (0.12), insula (0.08) and OFC (0.07), as well as to brainstem structures such as the NTS (0.12) and VTA (0.19). There was also relatively greater variation between subjects for the DRN than for any other structure analyzed, congruent with prior work on the serotonin system that has shown significant difference across individuals (Meneses and Liy-Salmeron, 2012; Gold, 2015). Interestingly, given the prior histologic evidence for LC-DRN and PAG-DRN connectivity (Hornung, 2003; Groves and Brown, 2005; Baker and Lui, 2020), we found a relatively low POC between these structures (0.02 and 0.05, respectively), which may reflect a limitation of our method for resolving extremely short-range connectivity within the brainstem (Figure 3). Taken together, our results provide important structural connectivity information, confirming significant connectivity between the DRN, amygdala, and hippocampus.

### Locus coeruleus

Studies from non-human primates have shown that the LC receives viscerosensory inputs from the NTS and DRN, as well as descending information from the amygdala, OFC, and rACC (Sara, 2009; Aston-Jones and Waterhouse, 2016). Recent work has also indicated that the LC plays a key role in shifting attention (Aston-Jones and Cohen, 2005; Bouret and Sara, 2005), emotionally salient memory formation and retrieval (Coull et al., 1999; Williams et al., 2000; Sterpenich et al., 2006), and cognition (Sara, 2009; Aston-Jones and Waterhouse, 2016). Notable findings in our study confirmed strong connectivity to the NTS (0.25), hippocampus (0.15), and the amygdala (0.27), but showed the greatest overall connectivity between the LC and the DLPFC (0.35) (Figure 4). This was significantly greater than LC-AMY, LC-HIPPO, or LC-NTS (for all, *p* < 0.0001) connectivity. This data supports electrophysiologic work in rats and non-human primates by demonstrating congruent anatomy in humans and supports the notion that a key role of the LC is to mediate attention, possibly through modulatory effects on the DLPFC, a cortical area known to become activated when subjects are asked to attend to specific stimuli (Sara, 2009; Aston-Jones and Waterhouse, 2016). Furthermore, these findings highlight work that demonstrates the importance of the LC in mediating attention and sympathetic activation during acute stress and response to threats, systems which have been shown to be maladaptive in anxiety and depressive disorders.

Additionally, the ability to use neuromelanin MRI sequencing has allowed for previous studies to explore the LC anatomy in a greater degree of detail compared to other brainstem nuclei. There is a high degree of anatomic correlation between our anatomic mask and neuromelain MRI sequences (note: neuromelain sequences were not used in this study) (Sasaki et al., 2008; Ogisu et al., 2013; Liu et al., 2017; Hämmerer et al., 2018).

### Nucleus tractus solitarius

The NTS is the major visceroafferent sensory nucleus for the vagal nerve complex (Henssen et al., 2019; Baker and Lui, 2020). It receives ~75% of afferent visceral sensory information and relays this information to other nuclei within the brainstem, namely the LC and DRN (Groves and Brown, 2005). While the NTS is involved in many physiologic functions including respiration and gastrointestinal regulation, it also has direct projections to the amygdala and has been implicated in panic disorder and memory formation (Clayton and Williams, 2000; Williams et al., 2000). Prior imaging studies have sought to identify the NTS on high resolution imaging and perform tractography from it (Henssen et al., 2019; Singh et al., 2019). However, differing anatomic definitions and the inherent difficulty of identifying the NTS on MRI, make meaningful comparisons challenging. Our data demonstrates significant connectivity between both the NTS and the amygdala (0.17) and NTS and the DLPFC (0.24), and we also show brainstem connections between the NTS and LC (0.26) and DRN (0.12) (Figure 5).

Given prior histological evidence, the expected findings of strong connectivity to the LC and DRN most likely indicate a pathway for afferent information from the vagus nerve to ascend to higher cortical centers (Chen et al., 1989; Groves and Brown, 2005; Groves et al., 2005). Interestingly, we also found a relatively high POC to the amygdala and the DLPFC. Studies in rodents have indicated that the NTS may be involved in fear memory formation *via* its connections with the amygdala (Clayton and Williams, 2000; Williams et al., 2000). Additional evidence supports that vagus nerve stimulation improves memory consolidation, and over 75% of vagal afferents project to the NTS (Hassert et al., 2004; Vanderah, 2019; Baker and Lui, 2020). Our results provide further evidence to support the anatomic basis of these findings in humans and could be used as the basis for further study of these phenomenon utilizing this atlas.

Clinically, the data on the NTS, DRN, and LC are interesting to consider in the context of existing work on vagus nerve stimulation (VNS). The NTS is thought to be the main vagal afferent nucleus for “body state information” (visceral sensory information from cardiopulmonary and digestive system) (Chen et al., 1989; Rutecki, 1990; Barnes et al., 2003; Henry et al., 2004; Groves and Brown, 2005; Groves et al., 2005; Fornai et al., 2011; Ruffoli et al., 2011). Based on animal and tract tracing studies, a large portion of fibers subsequently project to the LC and DRN which send adrenergic and serotonergic projections to cortical structures (Tucker et al., 2002; Angeles Fernández-Gil et al., 2010; Venkatraman et al., 2017). While it is not possible to delineate specific fiber types from this analysis, we found high POC between the LC-NTS (0.26), NTS-DRN (0.12), LC-AMY (0.27), and DRN-AMY (0.20) (Figures 3–5). Both the LC and DRN showed comparably strong connectivity to the DLPFC, Hippo, and Insula as well. Taken together, this atlas could prove useful to study the mechanism of VNS and possible structural reorganization of these pathways after a therapeutic intervention.

The NTS, in association with other autonomic nuclei, has been implicated in early stages of alpha-synucleinopathies that ultimately result in wide spread cognitive and neurodegenerative decline such as Parkinson's Disease (Del Tredici et al., 2002; Wang et al., 2020). Better methods of brainstem imaging could be useful in the future to help with earlier diagnosis of these disorders as the NTS, LC and other lower brainstem nuclei have been shown to be among the first affected by deposits of alpha-synuclein (Dickson et al., 2009; Seidel et al., 2014). The clinical relevance of these early histologic findings has yet to be fully described.

### Ventral tegmental area

The three main midbrain regions containing dopaminergic neurons are the retrorubral field, substantia nigra pars compacta, and VTA. The main dopaminergic system involved in limbic processes is the mesocorticolimbic pathway (Figure 1), which arises from the VTA and projects *via* the medial forebrain bundle to the NAc and prefrontal cortex (Alcaro et al., 2007; Arias-Carrión and Pöppel, 2007; Russo and Nestler, 2013; Beier et al., 2015; Settell et al., 2017). The VTA also receives feedback inhibition from the striatum, cortex, as well as the hippocampus and amygdala (Yim and Mogenson, 1982; Brog et al., 1993; Lu et al., 2005; Alcaro et al., 2007; Arias-Carrión and Pöppel, 2007; Beier et al., 2015). The VTA is involved in motivation, reward, and arousal, and the dopamine system is known to be affected in disease states such as Parkinson's Disease, addiction, depression, and schizophrenia (Kalivas, 1993; Russo and Nestler, 2013; Settell et al., 2017; Venkatraman et al., 2017; Anand et al., 2018).

In our analysis, the greatest POCs were between the VTA and the DLPFC (0.25), amygdala (0.22), hippocampus (0.17) and NTS (0.14) (Figure 6). Interestingly, despite the well-established relationship between the VTA and the NAc, we found low POC (0.02) between these two structures. This is likely because our methodology compares relative POC between target regions and, though we do control for it, can still be affected by the total number of fibers between a seed and target. Our findings regarding VTA-DLPFC connectivity and VTA-AMY connectivity are particularly notable as several DTI studies have previously linked the VTA with frontal lobe areas (namely the prefrontal cortex and OFC) but did not assess or demonstrate significant connectivity with temporal lobe structures (namely the amygdala and hippocampus) (Coenen et al., 2009, 2018; Anthofer et al., 2015; Hosp et al., 2019). Here, we found no significant difference between VTA-DLPFC or VTA-AMY POC (*p* = 0.26). This, combined with relatively low OFC POC (0.07) and a relatively high hippocampus POC (0.17), demonstrates a distinct connectivity pattern to *both* frontal and temporal lobe structures. The VTA is a potential target for deep brain stimulation as well as other neuromodulation techniques, and it is therefore highly important to consider its connectivity profile (Settell et al., 2017; Wang et al., 2018; Vyas et al., 2019). While dopaminergic connections between the VTA, amygdala, and hippocampus have previously been established, much of what is known is based on animal studies, and demonstrating this consistently in humans is of vital importance for the development of therapies based on this anatomy (Russo and Nestler, 2013; Beier et al., 2015).

### Periaqueductal grey

The PAG is a complex, heterogenous group of neurons that interacts with many brain regions and has roles in cardiorespiratory control, pain, fear, anxiety, and goal directed defensive behaviors (Ezra et al., 2015; Rozeske et al., 2018; Silva and McNaughton, 2019). Prior DTI studies have demonstrated PAG and prefrontal cortex connectivity. This is of interest because this connection has been suggested as a mechanism for the conscious modulation of pain signals (Sillery et al., 2005; Owen et al., 2008; Ezra et al., 2015; George et al., 2019; Silva and McNaughton, 2019). Furthermore, amygdala and insular connections have been demonstrated and are theorized to be involved in the emotional response to pain (Levy et al., 1987; Sillery et al., 2005; Owen et al., 2008; Ezra et al., 2015; Sims-Williams et al., 2017).

We find a non-selective pattern of connectivity with POC values above 0.10 for the OFC, insula, hippocampus, DLPFC and amygdala, confirming both the frontal and temporal connectivity patterns found in previous studies described above. The only brainstem region with significant connectivity was the DRN (0.10), consistent with the theory that these two regions encircling the aqueduct are closely linked and involved in the processing of aversive stimuli (Figure 7). While much of the data generated here was previously known, we sought to include the PAG in our atlas given its interplay with the DRN and importance in chronic pain.

### Modulation

Several attempts have been made to modulate these brainstem regions with varying degrees of success (Bittar et al., 2005; Bari et al., 2014; Akram et al., 2016; Wang et al., 2018; Vyas et al., 2019). By providing here an atlas from a large cohort of subjects, we hope this data can be potentially used to help target brainstem nuclei more effectively (and/or their upstream or downstream targets) in future trials of deep brain stimulation for neuropsychiatric disorders.

## Limitations

There are several important limitations to this study. There are inherent limitations described in detail regarding registration and tractography (Kinoshita et al., 2005; Thomas et al., 2014; Alhourani and Richardson, 2015; Schilling et al., 2019). The high-quality imaging protocols of the Human Connectome Project address several of the concerns related to artifact and scan acquisition (Van Essen et al., 2013). The brainstem in particular can be affected by arterial pulsations such as the basilar artery which result in a decrease in image quality. Some authors have attempted to correct for this motion, however we did not find significant artifact in our data so did not perform any correction (Krupa and Bekiesińska-Figatowska, 2015; Tang et al., 2018). The large scale of our study means that artifactual errors in individual scans have a minimal (<1%) impact on our results. Another limitation involves potential errors in anatomic masking. Care must be taken to accurately mask these brainstem structures as small anatomic errors, especially at early stages, can be compounded in the analysis. We attempted to address this by carefully creating each subject specific mask by hand (Figure 2), having multiple individuals including a neuroradiologist and neurosurgeon independently check each mask for accuracy, utilizing high quality scans and reference resources, and clearly defining our anatomic boundaries in this paper.

We also did not run whole brain tractography and may be missing fiber tracts that did not involve our preselected target regions. However, as we were interested specifically in limbic connectivity, especially for monoamine nuclei, we preselected ROIs based on the existing literature where we expected the highest anatomic connectivity, and this method allowed us to appreciate differences between specific regions of interest more clearly than whole brain tractography. It is also likely that some of the seed masks overlapped with large white matter tracts that abut these structures, this method prevented large motor and sensory tracts from complicating the analysis.

Given the small anatomical regions involved, some portions of the seed masks could overlap each other. The effect of this would be an over estimation of the connectivity variable between a given seed and target. It could also generate off target effects (in cases where adjacent nuclei may have been included in the seed mask), but should not affect the overall pattern of results for a single nucleis as each nucleus was run through tractography independently. Lastly, our method cannot determine directionality and care should be taken in ascribing any directional connectivity.

## Future directions

There are multiple alternative methods that would be useful to study brainstem connectivity. Here, we utilized 3T structural images, which provide good anatomic detail, but newer and more specialized techniques also have roles to play. For example, *in vivo* 7T high resolution imaging combined with novel methods such as track-density imaging has shown excellent results in delineating subcortical structures such as the thalamus (Basile et al., 2021). However, this data is difficult to generate. *Ex vivo* 7T imaging can also obtain higher resolution images for delineation of nuclei, however diffusion data from this is more difficult to interpret and is likely distorted by methods to prepare the tissue (Edlow et al., 2019; Roebroeck et al., 2019). Comparing the results in this atlas and determining if results hold across imaging modalities would be a useful next step. Additionally, we plan to perform whole brain tractography from these nuclei as a supplement to this existing analysis. Lastly, given that the Human Connectome Project has a significant amount of subject level behavioral and behavioral data available, we plan to study the correlation between structural connectivity and emotion-related behavioral traits. Future work is needed to elucidate if structural variation in individuals is associated with behavioral or personality traits.

## Conclusion

The brainstem is an essential component of the limbic system. Monoamine and other modulatory nuclei in the brainstem project widely to cortical and subcortical limbic regions and each has a specific pattern of connectivity. An understanding of this basic structural anatomy is a critical step in understanding disease processes, such as addiction, chronic pain, and depression and the development of novel therapeutics. Further studies are warranted to characterize the functional significance of the structural connectivity of each nucleus and the relationship of structural connectivity with neuropsychiatric symptoms.

## Data availability statement

The datasets presented in this study can be found in online repositories. The names of the repository/repositories and accession number(s) can be found in the article/Supplementary material.

## Author contributions

SL and AB conceived of the project. AB designed the original algorithm and oversaw all aspects of the projected. SL primarily prepared the manuscript, collected and analyzed the data, and created the figures. MM, AI, MJ, DA, and WW contributed to data acquisition and drafted portions of the manuscript. SH, JA-C, and TK provided review of anatomical accuracy and expertise in interpretation of results as well as review of the final manuscript. AH assisted with figure creation and provided expertise to use the data in the creation of a Lead-DBS atlas. All authors contributed to the article and approved the submitted version.

## Supplementary material

Anatomic supplement 1 Axial slices from the midbrain through the pons and medulla on an MNI 0.5 mm template brain with seed masks averaged across 197 subjects to demonstrate anatomic boundries of masks. Dorsal Raphe Nucleus: Pink; Periaquaductal Grey: yellow-red; Ventral Tegmental Area: blue; Locus Coreleus: yellow; Nucleus Tractus Solitaris: green.
