# Corrigendum: A structural connectivity atlas of limbic brainstem nuclei

**DOI:** 10.3389/fnimg.2024.1405806

**Published:** 2024-04-30

**Authors:** Simon Levinson, Michelle Miller, Ahmed Iftekhar, Monica Justo, Daniel Arriola, Wenxin Wei, Saman Hazany, Josue M. Avecillas-Chasin, Taylor P. Kuhn, Andreas Horn, Ausaf A. Bari

**Affiliations:** ^1^Department of Neurosurgery, David Geffen School of Medicine at the University of California, Los Angeles, Los Angeles, CA, United States; ^2^Stanford Department of Neurosurgery, Stanford University, Palo Alto, CA, United States; ^3^Department of Radiology, VA Greater Los Angeles Healthcare System, David Geffen School of Medicine at UCLA, Los Angeles, CA, United States; ^4^Department of Neurosurgery, University of Nebraska Medical Center, Omaha, NE, United States; ^5^Department of Psychiatry and Biobehavioral Sciences, University of California, Los Angeles, Los Angeles, CA, United States; ^6^Movement Disorder and Neuromodulation Unit, Department of Neurology, Charité–Universitätsmedizin Berlin, Corporate Member of Freie Universität Berlin and Humboldt–Universität zu Berlin, Berlin, Germany; ^7^Department of Neurology, Center for Brain Circuit Therapeutics, Harvard Medical School, Brigham and Women's Hospital, Boston, MA, United States; ^8^Massachusetts General Hospital Neurosurgery and Center for Neurotechnology and Neurorecovery (CNTR) at MGH Neurology Massachusetts General Hospital, Harvard Medical School, Boston, MA, United States

**Keywords:** brainstem, deep brain stimulation, limbic system, tractography, atlas

In the published article, there was an error in the legends for Figure 6 and Figure 7 as published. The captions were switched such that the caption for figure 6 was on figure 7 and vice versa. The corrected legends appear below.

Figure 6. Periaqueductal grey structural connectivity. **(A)** MNI space structural connectivity results visual representation averaged over all 197 subjects. Brighter yellow on heat map indicates a high number of samples passing through a given point that will eventually reach a target map (brighter yellow = more samples). Dark green: DLPFC; pink: OFC; brown: AMY; blue: HIPPO; purple: insula; orange: NAc; light green: rACC. **(B)** Mean connectivity results with dashed line showing mean and 95% CI, each point on graph shows result from individual subject. **(C)** Anatomic MNI mask of seed region.

Figure 7. Ventral tegmental area structural connectivity. **(A)** MNI space structural connectivity results visual representation averaged over all 197 subjects. Brighter yellow on heat map indicates a high number of samples passing through a given point that will eventually reach a target map (brighter yellow = more samples). Dark green: DLPFC; pink: OFC; brown: AMY; blue: HIPPO; purple: insula; orange: NAc; light green: rACC; **(B)** Mean connectivity results with dashed line showing mean and 95% CI, each point on graph shows result from individual subject. **(C)** Anatomic MNI mask of seed region.

The authors apologize for this error and state that this does not change the scientific conclusions of the article in any way. The original article has been updated.

